# Supplementary material for: Survival After Shunt Therapy in Normal-Pressure Hydrocephalus: A Meta-Analysis of 1614 Patients
Source: Neurol Int. 2024 Nov 11;16(6):107. doi: 10.3390/neurolint16060107 (PMC11587452; doi:10.3390/neurolint16060107)
Supplement: Supplementary file 1 [file neurolint-16-00107-s001.zip › Prisma Checklist Supplementary 1.pdf]

PRISMA 2020 Checklist

Section and Topic	Item #	Checklist item	Location where item is reported
TITLE			
Title	1	Survival after Shunt Therapy in Normal Pressure Hydrocephalus: A meta-analysis of 1614 patients	Title
ABSTRACT			
Abstract	2	BACKGROUND: Ventriculoperitoneal (VP) shunt therapy is a crucial intervention for normal pressure hydrocephalus (NPH). This meta-analysis delves into survival time and the impact of baseline symptom burden on survival after VP shunt therapy for NPH, employing reconstructed pooled survival curves and a one-stage meta-analysis.<br>METHODS: IPD regarding overall survival (OS) were acquired from published Kaplan-Meier charts, utilizing the R package IPDfromKM in R (Version 4.3.1, The R Foundation for Statistical Computing). Reconstructed Kaplan-Meier charts were then generated from the pooled IPD data. Both one-stage and two-stage meta-analyses were executed, with hazard ratios (HR) employed as metrics to evaluate effectiveness.<br>RESULTS: From the intial screening of 216 records, five articles encompassing 1614 patients met the eligibility criteria for inclusion. Two articles encompassing 1043, 1022, and 956 patients were included for the survival analyses stratified by gait score(1-4/>4), continence score (1-3/>3), and mRS (≤2/>2). Patients with good gait demonstrated a mean survival of 8.24 years, while those with poor gait had 6.19 years (log-rank test: $p < 0.001$). The HR for gait was 2.25 (95% CI: 1.81-2.81, $p < 0.001$). Continence score stratification revealed a significant difference in survival time (log-rank test: $p < 0.001$), with a HR of 1.66 (95% CI: 1.33-2.06, $p < 0.001$). Similarly, mRS stratification demonstrated a significant survival difference (log-rank test: $p < 0.001$), with a HR of 2.21 (95% CI: 1.74-2.80, $p < 0.001$). The reconstructed survival curves for all NPH patients treated with VP shunt therapy, pooling data from five studies, revealed a median survival time of 8.82 years (95% CI: 8.23-9.40). Survival rates at 1, 3, 5, 7, 9, 11, and 13 years were 95.7%, 83.8%, 70.5%, 59.5%, 48.7%, 35.8%, and 25.4%, respectively. Comparison with a general control population showed a HR of 1.79 (95% CI: 1.62-1.98, $p < 0.001$).<br>CONCLUSIONS: This comprehensive meta-analysis underscores the influence of baseline symptom burden on survival after VP shunt therapy in NPH. Therapy in the early stages for those without significant comorbidities may enhance survival.	Abstract
INTRODUCTION			
Rationale	3	However, the exact extent of clinically anticipated profit such as the longitudinal data of patients undergoing surgery for normal-pressure hydrocephalus remains subject of intensive investigation. Due to the chronic nature of this condition, a symptom-control might result in rise in quality of life and in case of gait disturbance/ataxia control even in higher overall performance and potentially also longer overall survival. Despite the fact that there are several retrospective/observational reports on long term outcome, a individual patient data meta-analysis has not been performed yet.	Introduction
Objectives	4	The aim of our work to analyze is to analyze the impact of baseline symptom burden on survival after NPH treatment via VP shunt surgery	Introduction
METHODS			
Eligibility criteria	5	We searched for "Normal Pressure Hydrocephalus" AND "Survival" or "Normal Pressure Hydrocephalus" AND "Mortality" in Pubmed, Medline, Cochrane and Embase database until 1st January 2024 and found 240 eligible studies. Subsequently, we screened meta-analyses on subarachnoid hemorrhage for further possibly eligible studies. Included were all studies describing rerupture-free survival data of the initially treated ruptured aneurysms ("target aneurysm") after Coiling or Clipping with Kaplan-Meier charts and corresponding patient number at risk tables. We excluded case studies, study protocols, non-clinical trials and meta-analyses. The rerupture event is limited to rebleedings from an already previously ruptured aneurysm which has been successfully treated by coiling or clipping. No data on rerupture form untreated aneurysms at other sites or de novo aneurysms that caused a second aSAH were included.<br>Two reviewers (JW, MV) independently screened abstracts, and full-text articles for two rounds, with any residual conflicts resolved by a third reviewer (EG).	Methods
Information	6	We searched for "Normal Pressure Hydrocephalus" AND "Survival" or "Normal Pressure Hydrocephalus" AND "Mortality" in Pubmed,	Methods

Section and Topic	Item #	Checklist item	Location where item is reported
sources		Medline, Cochrane and Embase database until 1st January 2024 and found 240 eligible studies.	
Search strategy	7	We searched for "Normal Pressure Hydrocephalus" AND "Survival" or "Normal Pressure Hydrocephalus" AND "Mortality" in Pubmed, Medline, Cochrane and Embase database until 1st January 2024 and found 240 eligible studies. Subsequently, we screened meta-analyses on subarachnoid hemorrhage for further possibly eligible studies.	Methods
Selection process	8	Excluded were all studies with other types of hydrocephalus and studies which did not reported on number at risk. Included were all two-arm and multi-arm studies reporting on OS of NPH patients. Control group was represented by a national registry. Survival was stratified based on gait score, continence score and mRS.	Methods
Data collection process	9	The data collection was performed by two authors independently (MV, JW). First the study titles were screened, then the corresponding abstract and in case of further uncertainty the full-text was screened by two authors (JW and EG) until all retrieved studies were either included or excluded. Two authors (JW, MV) independently extracted the following data from the studies: clinical and molecular characteristics of GB patients. We extracted and combined the IPD for overall survival using the Kaplan-Meier plots and the hazard ratio of the included studies. The data-reconstruction was performed with the R package *IPDfromKM* in R software version 4.3.1 (R Foundation for Statistical Computing, Vienna, Austria).	Methods
Data items	10a	We analyzed the studies to conduct a meta-analysis according to the following outcomes:<br>1) In case of VP-Shunt insertion for NPH following outcomes were analyzed: Probabilities of survival after VP-shunt surgery stratified by gait, continence, and mRS.	Methods
Study risk of bias assessment	11	The National Institutes of Health Quality Assessment Tool for observational cohort and cross-sectional studies (NIH-QAT) was used for the assessment of quality and risk of bias of included studies.	Methods
Effect measures	12	We measured and reported outcomes in Forest-plots, providing heterogeneity and inconsistency analysis, pooled hazard ratios and statistical significance. Furthermore, a pooled Kaplan-Meier chart of the reconstructed IPD was generated.	Methods
Synthesis methods	13a	To see all the eligible studies and reported outcomes, see Table 1	Methods
	13b	To see all the eligible studies and reported outcomes, see Table 1	Methods
	13c	For visualization of our meta-analysis, Forest Plots were created with the R package *Metafor*. IPD was generated using the R package *IPDfromKM* in R software version 4.3.1 (R Foundation for Statistical Computing, Vienna, Austria) [11]. Kaplan-Meier curves of rerupture-free survival were constructed for the whole included patient cohort using the R package *Survminer* and *Survival* in R software version 4.3.1 (R Foundation for Statistical Computing, Vienna, Austria).	Methods
	13d	Patient demographics and disease-specific characteristics of the included studies were recorded. The IPD information of all survival data from all the included trials was pooled, and Kaplan-Meier curves of overall survival were constructed for the whole included patient cohort using the R package *Survminer* and *Survival* in R software version 4.3.1 (R Foundation for Statistical Computing, Vienna, Austria). The 1-, 3-, 5-, 7-, 9-, 11- and 13-years survival probabilities were calculated. The hazard ratios (HR) of each individual study as well as the pooled HR and corresponding 95% confidence intervals (CI) between those with low and high preoperative symptom burden were estimated.<br>In the two-stage meta-analysis, we combined the estimated hazard ratios (HRs) and their corresponding 95% CI from individual studies using a random-effects model, employing the generic inverse variance method. The calculated HRs were logarithmically transformed (LN).	Methods

Section and Topic	Item #	Checklist item	Location where item is reported
		For each study, the standard error (SE) was derived from the 95% CI using the formula: SE = (LN(upper CI limit) – LN(lower CI limit)) / 3.92, as outlined in the Cochrane Handbook for Systematic Reviews of Interventions, Version 6.4. Heterogeneity across the included studies was evaluated using I^2 statistics, with a threshold of >50% displaying substantial heterogeneity. The aggregated results were presented in forest plots utilizing the R package *Metafor*. Weight of the relative contribution of the included studies, based on the sample size, was considered concerning the estimation of the treatment effects. A significance level of $p < 0.05$ was considered statistically significant. To evaluate publication bias, we created funnel plots with the R package *Metafor*. Visual assessment via funnel plots was performed.	
	13e	Not available	Methods
	13f	Not available	Methods
Reporting bias assessment	14	According to our strict inclusion criteria, we do not suppose to have bias due to missing results. Risk of bias assessment was performed for all included studies	Methods
Certainty assessment	15	Quality assessment was performed and the risk of bias assessment is summarized in supplemental figure 2.	Methods
RESULTS			
Study selection	16a	Process of the search and selection is summarized in Figure 1.	Figure 1
	16b	Included studies are summarized in table 1.	Figure 1
Study characteristics	17	Characteristics of included studies are summarized in table 1.	Table 1
Risk of bias in studies	18	Risk of bias analysis is reported in supplementary figure 2.	Supplemental Figure 2
Results of individual studies	19	Analysis of each outcome are separately reported on with it´s own forest plot in Table 2 and Figure 4.	Figures 4 and table 2
Results of syntheses	20a	Risk of bias assessment is summarized in the supplementary figure 2. Figure 5 represents the funnel plot visualizing the publication bias	Suppl. Figure 2 & Figure 5
	20b	Results of the statistical syntheses are summarized in the Figures 2, Figure 3, and Figure 4.	Figures 2-4
	20c	Results regarding heterogeneity are presented in the Figure 4.	Figure 4
	20d	Presented	Results
Reporting biases	21	Risk of bias and publication bias assessment is shown in supplementary figure 2 and figure 5	supplementary figure 2 & Figure 5
Certainty of evidence	22	Not available	NA
DISCUSSION			
Discussion	23a	Provide a general interpretation of the results in the context of other evidence.<br>Presented	Discussion

PRISMA 2020 Checklist

Section and Topic	Item #	Checklist item	Location where item is reported
	23b	Discuss any limitations of the evidence included in the review.<br>Presented	Discussion, last paragraph
	23c	Discuss any limitations of the review processes used.<br>Presented	Discussion, last paragraph
	23d	Discuss implications of the results for practice, policy, and future research.<br>Presented	Discussion
OTHER INFORMATION			
Registration and protocol	24a	The study was registered in the "International Prospective Register of Systematic Reviews" (PROSPERO) in 2023 (Submission-ID:…)	Methods
	24b	The study was registered in the "International Prospective Register of Systematic Reviews" (PROSPERO) in 2023 (Submission-ID:...)	Methods
	24c	No amendments to describe	
Support	25	There is no financial conflict of interest to declare	NA
Competing interests	26	No competing interests of the authors to delcare	NA
Availability of data, code and other materials	27	Template data collection form, data extracted and used for the analysis are reported in the section "methods". The R packages (IPDfromKM, SURVMINER, METAFOR, and SURVIVAL) are available online	Methods

From: Page MJ, McKenzie JE, Bossuyt PM, Boutron I, Hoffmann TC, Mulrow CD, et al. The PRISMA 2020 statement: an updated guideline for reporting systematic reviews. BMJ 2021;372:n71. doi: 10.1136/bmj.n71

For more information, visit: http://www.prisma-statement.org/
