# Supplementary material for: Survival After Shunt Therapy in Normal-Pressure Hydrocephalus: A Meta-Analysis of 1614 Patients
Source: Neurol Int. 2024 Nov 11;16(6):107. doi: 10.3390/neurolint16060107 (PMC11587452; doi:10.3390/neurolint16060107)
Supplement: Supplementary file 1 [file neurolint-16-00107-s001.zip › Supplementary 2 - NIH Quality assessment.pdf]

NIH Quality assessment **Observational cohort and Cross-sectional** studies	**Research question or objective stated**	**Study population defined**	**Participant rate at least 50% from eligible**	**Subject recruitment and eligibility criteria**	**Sample size justification**	**Exposure(s) measured prior to the outcome(s)**	**Sufficient time frame**	**Examination of different levels of exposure as related to the outcome**	**Definition and validation of the exposure measures**	**Exposure(s) assessed more than once**	**Definition of outcome measures**	**Blinded assessors**	**Loss to follow-up after baseline of 20% or less**	**Statistical measure and adjustment of key confounding variables**
**Andrén et al 2020 [14]**	**Y**	**Y**	**Y**	**N**	**NA**	**N**	**Y**	**N**	**Y**	**N**	**Y**	**N**	**Y**	**Y**
**Andrén et al. 2021 [21]**	**Y**	**Y**	**Y**	**N**	**NA**	**N**	**Y**	**N**	**Y**	**N**	**Y**	**N**	**Y**	**Y**
**Eklund et al. 2023 [22]**	**Y**	**Y**	**Y**	**N**	**NA**	**N**	**Y**	**N**	**Y**	**N**	**Y**	**N**	**Y**	**Y**
**Pyykkö et al. 2018 [23]**	**Y**	**Y**	**Y**	**N**	**NA**	**N**	**Y**	**N**	**Y**	**N**	**Y**	**N**	**Y**	**Y**
**Klinge et al. 2023 [24]**	**Y**	**Y**	**Y**	**N**	**NA**	**N**	**Y**	**N**	**Y**	**N**	**Y**	**N**	**Y**	**Y**

Y = Yes; N = No; NA = Not applicable
