# Supplementary figures and images for: Survival After Shunt Therapy in Normal-Pressure Hydrocephalus: A Meta-Analysis of 1614 Patients
Source: Neurol Int. 2024 Nov 11;16(6):107. doi: 10.3390/neurolint16060107 (PMC11587452; doi:10.3390/neurolint16060107)

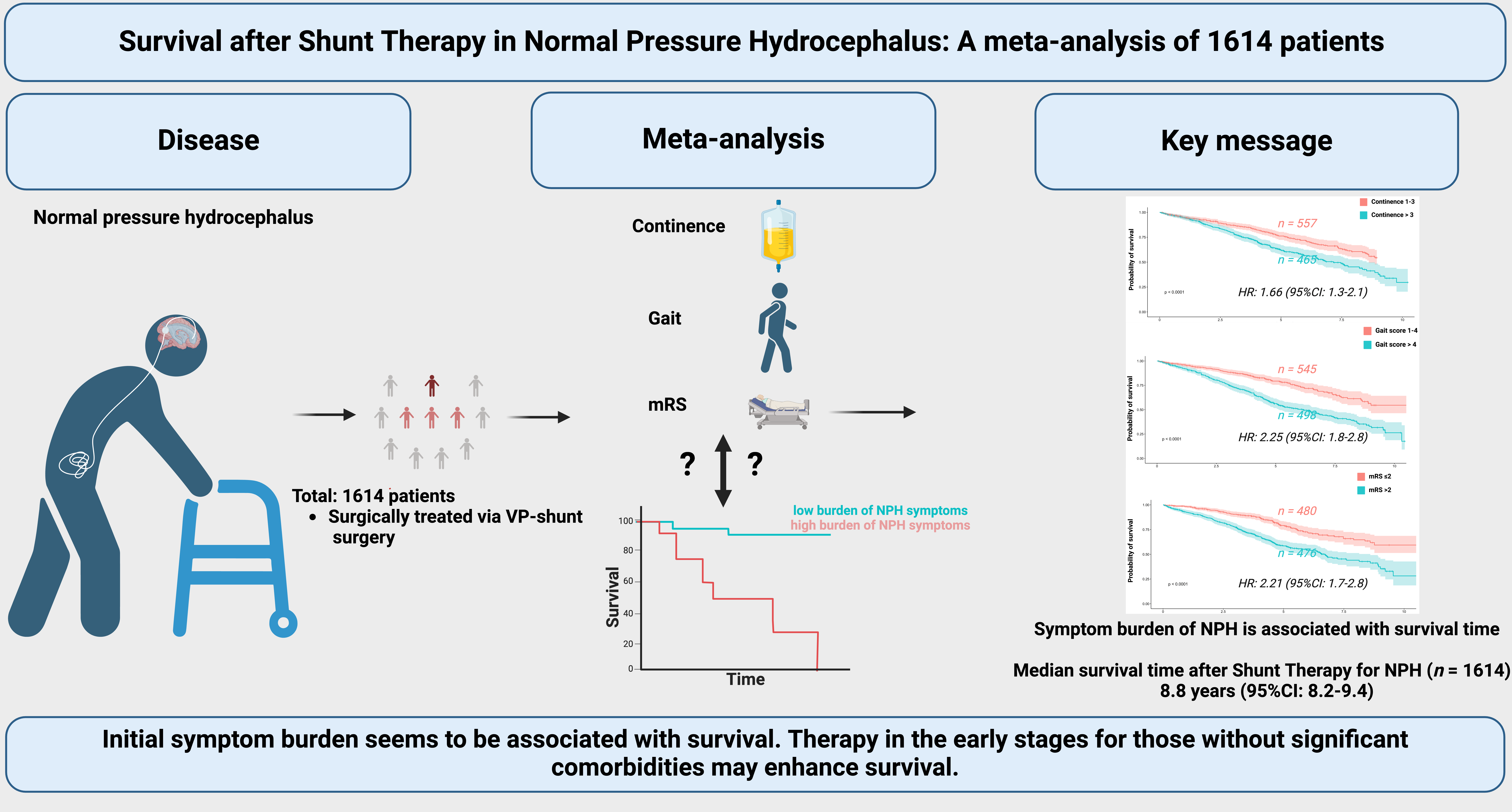

Supplement: Supplementary file 1 [file neurolint-16-00107-s001.zip › supplementary_figure_3_NPH.png]

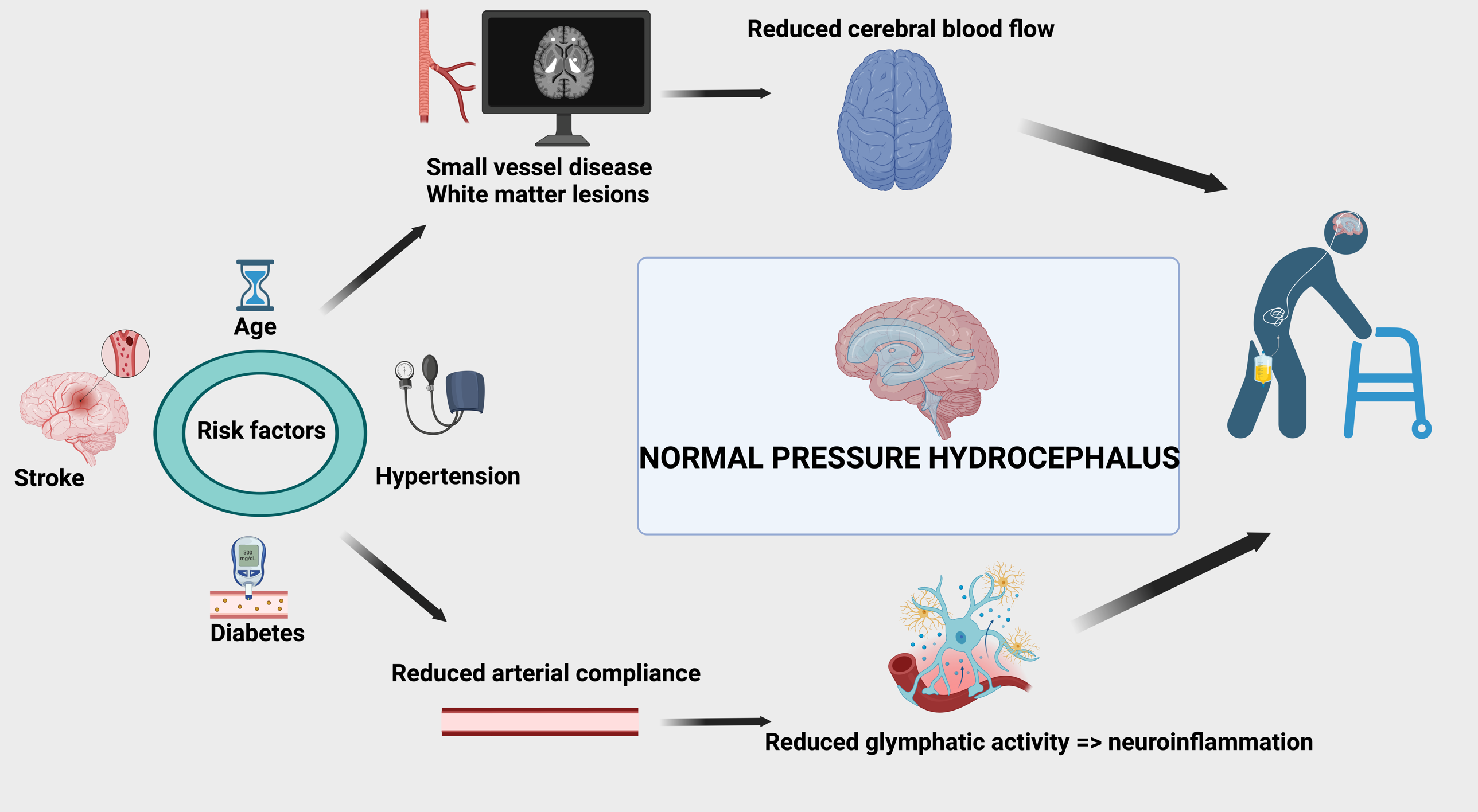

Supplement: Supplementary file 1 [file neurolint-16-00107-s001.zip › supplementary_figure_S4.png]
